# Congener-Specific Emissions from Floors and Walls Characterize Indoor Airborne Polychlorinated Biphenyls

**DOI:** 10.1021/acs.estlett.3c00360

**Published:** 2023-08-21

**Authors:** Moala K. Bannavti, Rachel F. Marek, Craig L. Just, Keri C. Hornbuckle

**Affiliations:** Department of Civil and Environmental Engineering, IIHR−Hydroscience & Engineering, University of Iowa, Iowa City, Iowa 52242, United States

**Keywords:** Atmospheric chemistry, Polychlorinated biphenyls, Gas chromatography mass spectrometry, Emissions, Materials, Aroclors

## Abstract

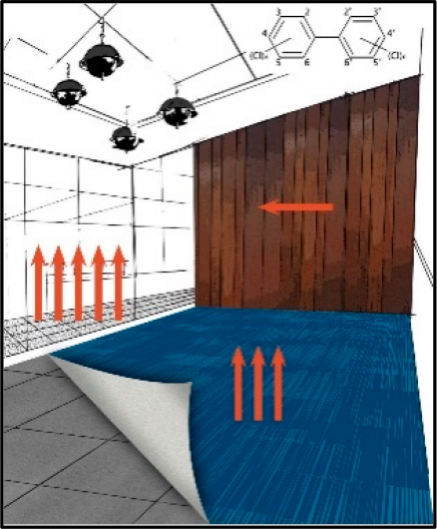

To reconcile the federal regulation of material polychlorinated biphenyl (PCB) concentrations with recently implemented state regulations of airborne PCBs, there is a need to characterize the relationship between PCB emissions from surfaces and air concentrations. We hypothesized that the magnitude and congener distribution of emissions from floors and walls fully account for the airborne PCBs measured in rooms constructed during the height of PCB production and sales. We measured emissions of PCB congeners from various wall and floor materials using polyurethane foam passive emission samplers before and after hexane wiping. Our results revealed that PCB emissions from flooring adequately predicted the magnitude and congener distribution of PCBs observed in the room air. Emissions varied by material within a single building (5 × 10^3^ ng m^–2^ day^–1^ from wood panel walls to 3 × 10^4^ ng m^–2^ day^–1^ from vinyl tile) and within the same room. Yet congener distributions between material emission PCB profiles and room air PCB profiles were statistically similar. Hexane wiping significantly reduced PCB emissions (>60%), indicating the importance of surface films as an ongoing source of airborne PCBs. The magnitude and congener distribution of material bulk concentrations did not explain that of material emissions or air concentrations. Passive measurements of polychlorinated biphenyl emissions from floors in a university building predict the concentrations of PCBs in room air.

## Introduction

Gas-phase emissions of polychlorinated biphenyls (PCBs) from PCB-containing building materials are sources of indoor airborne PCBs.^1−3^ Inhalation of these anthropogenic compounds may cause cancer, hormone disfunction, and cognitive learning disorders.^4^ In 1976, the U.S. Environmental Protection Agency banned the intentional manufacture and sale of PCBs.^5,6^ Prior to the ban, Aroclor PCBs were added to building materials such as tile adhesive, window caulking, and florescent light ballasts.^5,7^ These historic mixtures of PCBs still exist in the built environment, including homes, schools, and other public buildings.^3,7−16^ However, the documented history of PCB use in building materials and during building remodels is incomplete. Primary sources of airborne PCBs indoors can be difficult to find due to volatilization and deposition within a room.^17,18^ Thus, remediation efforts often include costly nontargeted source testing and analysis.

In the U.S., there is no federal regulation requiring the remediation of indoor airborne PCBs. Only building materials with PCB concentrations of 50 ppm and above require abatement from an indoor environment. The Code of Federal Regulations defines the protocol for the removal and disposal of the PCB source material. But no official process exists for identifying source materials and associated emissions’ relationships to airborne PCB concentrations.^19,20^ In this study, we used direct measurements of gas-phase emissions of specific materials and passive air samplers to characterize sources of PCBs to room air. Multiple materials can be PCB sources and sinks in a room, and an accurate inventory of associated emission sources can inform targeted remediation strategies.

We hypothesized that floors and walls were sources of airborne PCBs in a room constructed during the PCB era. Furthermore, we hypothesized that the magnitude and congener distribution of emissions from floors and walls fully account for the airborne PCBs in the room. To test these hypotheses, we measured surface emissions and room air concentrations for 209 PCB congeners in rooms without PCB-containing light ballasts or window caulking. Mass balance calculations linked the emissions of each congener to the concentrations of each congener in air. We used nonparametric statistical tests to evaluate the differences in congener distributions measured in the emissions and air. Using these two approaches, we determined the influence of PCB emissions from specific surfaces on the airborne concentrations in the rooms.

## Methods and Materials

### Site Description

All samples were collected between 2020 and 2022 from four rooms within the University of Iowa’s Institute of Rural and Environmental Health office and laboratory building constructed between 1972 and 1980 (Table S1). Three rooms had individual, packaged terminal air conditioner units that received outdoor air but were off during sampling. A fourth room was connected to a central air system that served multiple rooms. These rooms were unoccupied for three years prior to the study and contained an assortment of desks, chairs, and metal or wooden cabinetry. Each room had carpet laid on top of the same vinyl tile that was bare in the hallway. University records indicate that the floor and wall materials were installed in the 1970s. No information was available regarding when the plywood panel walls and floors were last cleaned. PCB-containing window caulking was removed from the east wing of the building in 2013 with retesting every year from 2013 to 2016. All PCB light ballasts were removed by 2013.

### Sampler Deployment

We collected and analyzed 12 airborne PCB samples using Harner-style double dome polyurethane foam passive air samplers (PUF–PAS) for 42 days hung from the ceiling tiles (∼2 m), 4 airborne PCB samples using low-volume air samplers for 2 days, 30 gas-phase emission samples using polyurethane foam passive emission samplers (PUF–PES) for 23 days, 15 instantaneous wipe samples, and 11 bulk material samples using methods as described in previous studies and the Supporting Information (SI).^17,21−23^ Congener-specific octanol-air partitioning coefficients (*K*_oa_) and effective sampling volumes (*V*_eff_, m^3^) for the PUF–PAS were calculated previously (eqs S1–S5).^24,25^ PUF–PES emission samplers capture gas-phase emissions on PUF as previously described.^14,26^ We deployed PUF–PES in five locations in triplicate including tile overlaid with carpet, wood panel, and hallway tile. We repeated the deployment of PUF–PES in the same locations (triplicate) immediately after a standard wipe test^27^ of all three materials, using 2 mL of hexane per wipe, to evaluate emissions after removal of surface PCBs (see SI). We collected five samples of carpet and six samples of wood panels for PCB analysis using a box cutter (Table S2). The wood panel consisted of plywood attached to cinder blocks with an adhesive. Gas chromatography tandem mass spectrometry (Agilent 7000 Triple Quad with Agilent 7890A GC and Agilent 7693 autosampler) in multiple reaction monitoring mode was used for identification and quantification of 209 PCBs as 171 chromatographic peaks (Table S3). The details of our sample extraction and instrument analysis can be found in the SI.

### Quality Assurance, Quality Control, and Statistical Analyses

Accuracy of our methods was assessed using analysis of certified PCB concentrations in NY/NJ sediment sprinkled on PUF (NIST Standard Reference Material 2585) (Figure S1). Accuracy was also assessed using an extraction of Aroclor 1016 to verify the appropriateness of surrogate recovery correction for lower-chlorinated congeners (Figure S2). Sample representativeness and reproducibility were assessed by placing triplicates of samplers at each location, side-by-side. Representativeness of the intended environment was also measured in the analysis of method blanks which were used to calculate the limits of quantification (Tables S4–S10). Precision of our sampling techniques and mass results were assessed with surrogate standard recoveries (Figure S3). Representativeness and accuracy were assessed by measuring emissions using PUF–PES over foil (negative control) in a room with a high PCB concentration to evaluate uptake of room air (Figure S4). Comparability was assessed with previous and concurrent measurements of PCBs in the rooms using other methods.^10,14,17,25,26^ We evaluated the differences in congener profiles using cosine theta analysis (cos θ).^17,25^ Cos θ varies from 0 (no correlation) to 1 (complete correlation; see SI). In addition to cos θ, we used the Wilcoxon signed rank (eq S6)^28^ test coupled with the Bonferroni Correction, mixed effects models, and random effects models to determine if congener distributions and masses among differing sample types could account for distributions and mass of PCBs observed in room air.

### Modeling

We used a mass balance equation to determine the amount of time needed for the PCB mass from targeted sources and the mass of PCBs found in room air to reach a steady state.
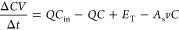


*C* is the concentration in the room at time *t* (ng m^–3^), *C*_in_ is the concentration of PCBs entering the room from the outdoors (ng m^–3^), *V* is the volume of the room (m^3^), and *Q* is the flow of air into and out of the room (m^3^ d^–1^). *E*_T_ is the total of all area-specific emission rates or the sum of emissions from tile overlaid with carpet and wood panel walls (ng m^–2^ d^–1^) multiplied by the area of the respective surface (m^2^) multiplied by the emissions from both flooring and walls by their respective surface areas to yield the total emissions from the surfaces per day (ng d^–1^). Deposition to all surfaces (ng m^–2^ d^–1^) is a product of the total surface area of the room (m^2^), *A*_s_; the deposition velocity (m d^–1^), *v*; and the concentration, *C*, at time *t*. We assume there is no PCB mass in the room at the commencement of the experiment (*C* = 0). When solving for *C*, the yield is
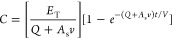


The airflow, *Q*, was derived from multiplying the area under the door (6.1 × 10^–3^ m^2^) with the average windspeed in the room measured by a 3D sonic anemometer (2.0 and 1.8 m s^–1^ for Rooms 132 and 137, respectively). We estimated the volume and surface area of Rooms 132 and 137 by adjusting the values reported here to that of a furnished space (0.93 for volume and 1.80 for surface area).^29^ We estimated the deposition velocities for particle size fractions between 1 and 2.5 μm, which includes PCBs (1.2 and 3.2 × 10^–4^ m h^–1^ in the vertical downward and horizonal direction, respectively), averaged these values, and multiplied the product by 24 (hours d^–1^) to derive a deposition velocity, *v*, of 14.4 m d^–1^. We conservatively assumed that PUF in furniture acted as a sink for 20% of emissions.^30,31^ Thus, we multiplied the emission rate from flooring by 0.8 before multiplying it by the surface area of the floor and adding the product to the wood panel wall to yield *E*_T_. Then we solved for *t* (days). All data from this study are published in Iowa Research Online^32^ (https://doi.org/10.25820/data.006187).

## Results and Discussion

### Material Measurements

**Figure 1:**
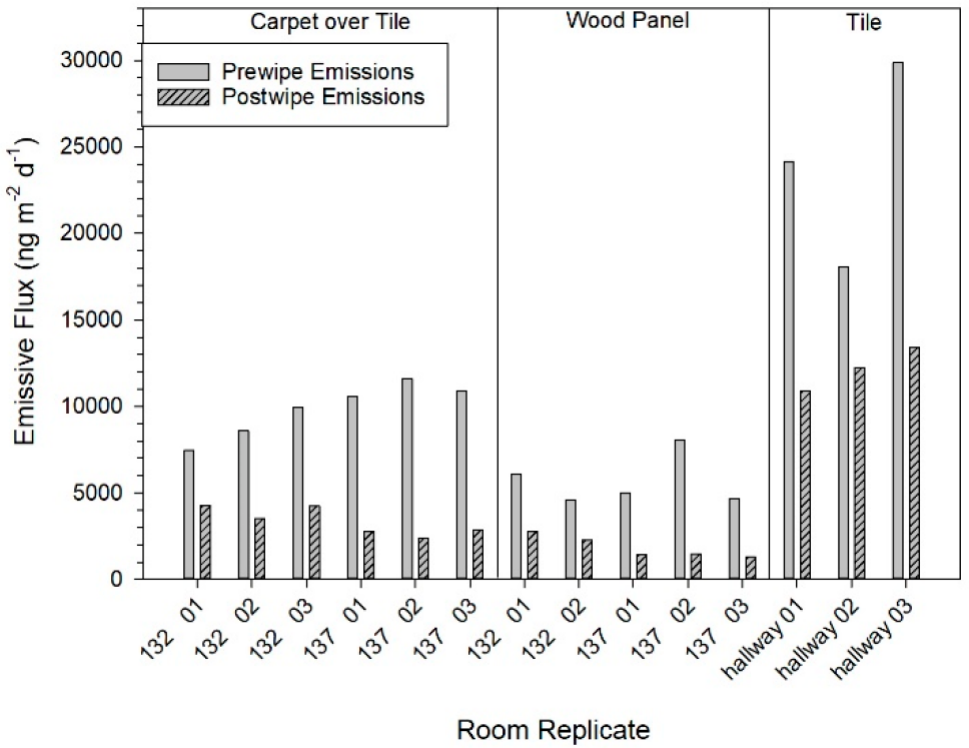
Total emissions of 205 PCBs from hallway tile, wood panel, and tile with carpet overlay before and after wiping with hexane and gauze.

We found PCB emissions from bare vinyl tile in the hallway to be significantly higher than emissions from tile overlaid with carpet and wood panel, both before and after hexane wiping (Figure 1). Before wiping, hallway tile emissions were about two and four times higher than tiles overlaid with carpet and wood panel, respectively. Tile overlaid with carpet had statistically higher emissions than wood panel before (*p*-value = 6.92 × 10^–4^) and after (*p*-value = 6.35 × 10^–5^) wiping regardless of room. Emissions of PCBs from hallway tile, tile overlaid with carpet, and wood panel walls before wiping were about 100 times higher than emissions previously reported from similar surfaces in residential apartments and from paint colorants using the same sampling apartus.^14,26^ Across 14 pairs of prewipe and postwipe PUF–PES, hexane wiping significantly reduced emissions by an average of 61 ± 14% (*p*-value = 1.2 × 10^–4^). The differences in the congener emissions before and after wiping were statistically significant. Most but not all (59%) PCB congener emissions were significantly reduced by hexane wiping.

The carpet and walls in the rooms contained PCBs (Figure S5). The largest individual bulk concentration was 1.2 × 10^4^ ng g^–1^ (12 ppm) from a carpet sample, and the lowest individual bulk concentration was from a wood panel sample, 319 ng g^–1^ (0.32 ppm). Carpet contained a significantly higher PCB mass per mass of material than wood panel regardless of location as per a mixed effects model (*p*-value = 4.06 × 10^–8^).

Room concentrations of ∑PCB in air ranged from 22 to 133 ng m^–3^ across all four rooms (Table S11). These indoor concentrations are 1 to 2 orders of magnitude higher than outdoor measurements reported from Persoon et al. (1.65 ng m^–3^) at the same location.^33^ The differences in air concentration from room to room were not statistically significant (*p*-value = 0.79), unlike our recent finding in a rural Iowa school that found room-to-room statistical differences in airborne PCB concentrations.^17^ The airborne PCBs measured in this current study (<133 ng m^–3^) were lower than or equal to levels that Herrick et al. reported in U.S. university buildings with historic caulk contamination (111–393 ng m^–3^).^34^ Air concentrations here were lower than those reported in Danish apartments (2.3 × 10^3^ ng m^–3^) and German schools (∼4.0 × 10^3^ ng m^–3^) with historic contamination of caulk sealants constructed during the same period that had not undergone remediation.^35−37^

The Code of Federal Regulations only considers a building material bulk PCB waste if the concentration is above 50 ppm (5 × 10^4^ ng g^–1^), even if there are high air concentrations or material emissions in the same room.^20^ Though the regulation requires removal at 50 ppm or greater, our findings suggest that surface materials less than 50 ppm or 5 × 10^4^ ng g^–1^, such as our wood panel and carpet, can still emit PCBs at a rate that produces air concentrations over 100 ng m^–3^. After consideration of PCB’s cancerous and noncancerous toxicity, Vermont state recently passed a law requiring testing of PCBs in all school rooms built or renovated during the PCB mass production era.^38^ The Vermont Department of Environmental Conservation determined that schools should take action to remediate rooms with 30–100 ng m^–3^ of PCBs depending on the students’ ages.^39^ All rooms measured in this study had concentrations within or above this range. Vermont further determined that schoolrooms with an air concentration of 90–300 ng m^–3^ must immediately cease occupancy.^39^ The room air concentrations resulting from source emissions in this study would require immediate action in Vermont schoolrooms, even though source bulk concentrations may be lower than 50 ppm or 5 × 10^4^ ng g^–1^.

We found that PCBs in the dust wiped off carpet over tile, wood panel, and bare tile surfaces (Table S12). The surface area of each wiped location was the same as that of one PUF disk: 1.53 × 10^–2^ m^2^. The mass of PCBs on each wipe ranged from 176 ng (carpet) to 1324 ng (hallway tile). Anderson et al. reported an average of 9.64 × 10^4^ ng m^–2^ of wiped PCBs from walls in Danish apartments which was about two times higher than that measured in this study (4.29 × 10^4^ ng m^–2^).^35^ On average, the PCB mass instantaneously wiped off material surfaces was equivalent to ten times the mass emitted in 1 day. However, the mass emitted postwiping during the same length of time was only one-half to one-fourth the mass emitted prior to wiping.

### Sources and Sinks

All surface wipe measurements, regardless of material or location ranged between 10^4^ and 10^5^ ng m^–2^. We found no significant difference between the masses wiped off carpet and wood panel regardless of location (*p*-value = 0.22) suggesting an evenly distributed removable surface PCB layer throughout all rooms.

**Figure 2:**
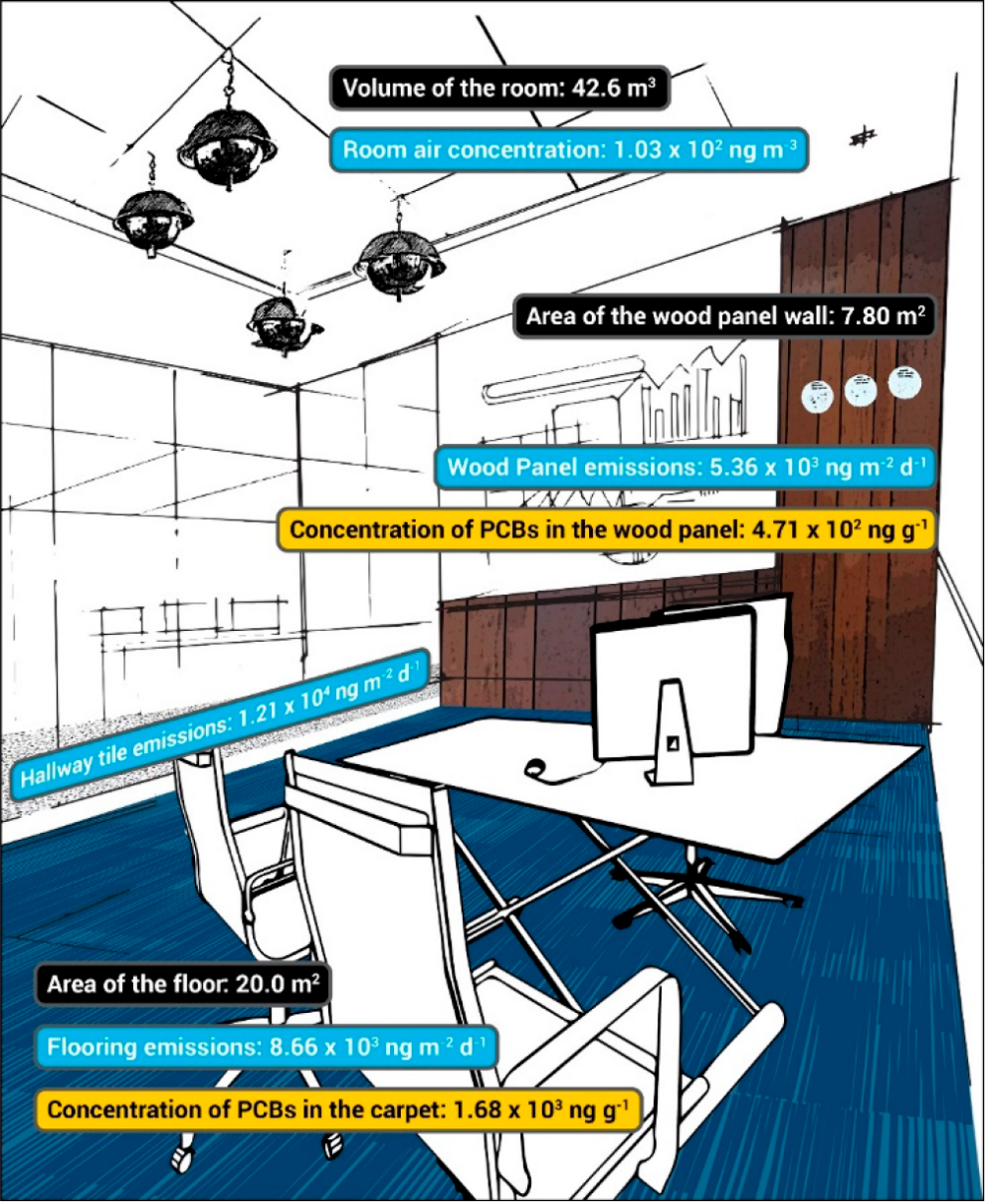
A sketch of Room 132 PCB emission sources where room volume and surface areas are in black, average airborne PCB concentration and material PCB emissions prewiping are in light blue, and material bulk concentrations are in yellow.

The PCB emissions measured in this study explain the concentrations of airborne PCBs in the rooms. We evaluated the prewipe PCB emission magnitude from floors and walls with the magnitude of airborne PCBs using a mass balance equation to determine the time to steady state (Figure 2).

We found at the rate of emissions measured before the materials were wiped with hexane, room air would reach the steady state value we measured in about 1 h for both Room 132 (79 ng m^–3^) and Room 137 (102 ng m^–3^). Emissions from walls and flooring after removal of the surface film indicate long-term absorption of airborne PCBs into the bulk material and/or diffusion of PCBs from underlying materials, including the adhesive under the tile.

In addition to calculating the time to steady state, we also estimated the time to depletion of the PCB reservoir in floors and walls provided emissions were constant over time, using the bulk concentration of each sample (ng m^–3^), total volume, and surface area of the material in the room and the emission rate of the material (ng m^–2^ days^–1^). At the measured postwipe emission rate, the PCBs we measured in wood panel walls from Room 132 and Room 137 would deplete in a few years. This is consistent with deposition and absorption from 50 years of exposure to elevated airborne PCBs since the building was constructed. We conclude that the wood panel wall is a secondary source of airborne PCBs. Emissions from the vinyl tile and overlying carpet are much higher and are likely to be primary sources, probably due to Aroclors added to adhesives used in flooring construction. For example, at the measured postwipe emissions rates, the bulk PCB concentration we measured in in carpet from Room 132 and Room 137 would require decades to deplete. We did not evaluate depletion rates for the tile because we could not completely separate it from the adhesive binding it to the building foundation.

### Congener Similarities

To determine if the congener profiles of material emissions are statistically similar to those of the congener distributions of the room air concentrations, we conducted three types of cos θ tests: one comparing room air profiles to vaporized Aroclor profiles, one comparing room air profiles to material emission profiles, and one comparing material bulk concentration profiles to material emissions profiles and room air profiles. Material emissions profiles were a mixture of Aroclor 1254 and Aroclor 1260 (Figure S6) which align with Aroclors used in caulking and other sealants in the 1970s when the rooms were constructed.^18^ For most rooms, the airborne PCB congener profile was most similar to the emissions from the hallway tile (cos θ > 0.97) (Table S13). In one room, the airborne PCB congener signal was most like that of the carpet emission signal from an adjacent room. In all rooms, congener profiles from wood panel emissions did not have a strong resemblance to room air congener profiles.

Unlike PCB congener distributions from material emissions, congener distributions from bulk material measurements were not as similar to congener distributions from room air. For example, emissions from tile overlaid with carpet had congener distributions similar to those in the room air (cos θ = 0.98) (Figure S7). Yet the PCB congener distributions from carpet itself in both rooms had a poorer correlation to that of the respective room air profile (cos θ = 0.86). This data reaffirm that the bulk concentrations are less useful in assessing PCB sources in a room than emissions sampling.

## Implications

Direct measurement of PCB emissions is a better indicator of important sources of airborne PCBs than solid material analysis. This finding suggests the need for a revision of federal statutes regulating the remediation of building materials indoors to focus on coupling room air concentrations with material emission rates as a method for source identification and reduction rather than bulk concentration. Our results support the characterization of material PCB emissions indoors for noninvasive and more targeted source identification, reducing the number of tests required to remediate and thus total costs.

## Supporting Information Available

Additional details on site description, material parameters, experimental methods, air sampler models, and quality control and assurance calculations including tables, graphs, equations, and figures of sample replicates and their statistical analysis (PDF)
