# Supplementary material for: Congener-Specific Emissions from Floors and Walls Characterize Indoor Airborne Polychlorinated Biphenyls
Source: Environ Sci Technol Lett. 2023 Aug 21;10(9):762–7. doi: 10.1021/acs.estlett.3c00360 (PMC10501191; doi:10.1021/acs.estlett.3c00360)
Supplement: Supplementary file 1 — ez3c00360_si_001.pdf [file ez3c00360_si_001.pdf]

Supporting Information

CONGENER-SPECIFIC EMISSIONS FROM FLOORS AND WALLS CHARACTERIZE INDOOR AIRBORNE PCBs

Authors. Moala K. Bannavti, Rachel F. Marek, Craig L. Just, Keri C. Hornbuckle *

Department of Civil and Environmental Engineering, IIHR-Hydroscience & Engineering
University of Iowa, Iowa City, Iowa USA 52242

*Corresponding author keri-hornbuckle@uiowa.edu Ph (319 384-0789)

Data generated in this research is available at DOI: https://doi.org/10.25820/data.006187.[1]

Number of pages: 29

Number of tables: 13

Number of figures: 7

Methods and Materials

Site Description

Rooms 132, 137 and 140 had floor, wall, and ceiling total surface areas of about 78 m^2. One hallway connected Rooms 132, 137, 140. All rooms had textured, lay-in tile drop ceilings. Rooms 132, 137, and 140 had three brick walls, painted white, and one wood panel wall. Room 171 had one brick wall and three sheetrock walls, painted light blue. There have been no reports of PCBs in the brick and mortar used to construct the building.

This building has historic contamination of Aroclor PCBs which were found in surface wipe, air, and caulk samples analyzed using NIOSH 5503 Mod. and EPA method 3540C/8082A. Remediation of the building in 2013-15 included replacement of PCB-containing light ballasts and window caulking.

Table S1. The construction date, dimensions, and building materials in each room. All rooms were 2.13 m tall.

Room	Construction Date	Surface Area floor (m^2)	Surface Area wood panel wall (m^2)	HVAC Type	HVAC On v Off	Additional Description
132	1972	19.97	7.8	Independent Unit	Off	Same hallway as Rooms 137 and 140
137	1972	19.49	7.7	Independent Unit	Off	Opposite Room 140
140	1972	19.37	7.7	Independent Unit	Off	Opposite Room 137
171	1980	10.86	N/A	Central Air	On	Separate wing from all other Rooms

Sample Collection and Extraction

The PUF-PAS were placed hanging from the rooms' ceilings. The circular PUF disk have a surface area of 0.0153 m^2 and depth of 0.015 m (Tisch Environmental, Cleves, OH, Part # TE-1014). All samples were handled using nitrile gloves. Before deployment, PUF were cleaned with 1:1 hexane–acetone. After drying under N_2, we wrapped each PUF individually in aluminum foil and stored them at −4 °C. We placed PUF–PAS in each location in quadruplet except for Room 171, where they were placed in triplicate. We deployed the PUF-PAS for six weeks (December 16, 2020, to January 28, 2021). Upon collection, PUF were wrapped in the same aluminum foil from deployment. The foil was stored in zip lock bags at −4 °C during deployment. Congener-specific effective sampling volumes (V_{eff}, m^3) were calculated as a function of the partitioning coefficient (K_{PUF}, unitless), the volume of the PUF (V_{PUF}, m^3), the deployment time (t, d), and the sampling rate (R_s, m^3 d^{-1}) in **Equation S1**.[2]

$$V_{eff} = (V_{PUF}K_{PUF})[1 - e^{-\left(\frac{R_S}{V_{PUF}K_{PUF}}\right)t}] \qquad \textbf{Equation S1}$$

$$R_S = \left(f_{on}\sqrt{WS_{on}} + f_{off}\sqrt{WS_{off}}\right)\left(\frac{1}{\sqrt[3]{MW}}\right)10^{[0.0012T+c]}$$ **Equation S2**

The indoor R_s is a function of the molecular weight of the compound (MW, g mol^{-1}), the air temperature (T, °C), the fraction of the day when ventilation is on or off (f_{on}/f_{off}), the windspeed when the ventilation is on or off (WS_{on}/WS_{off}), and the empirical constant of double-dome Harner-style samplers (c) in **Equation S2**. The temperature inside the rooms was 22 °C year-round.

We used a 3D sonic anemometer to measure the wind speed in each room, including the location directly below the PUF-PAS deployments. The HVAC system was manually turned off in Rooms 132, 137 and 140 ($f_{off} = 1$) throughout all sampling. In all rooms the empirical constant for double-dome samplers, c, is 1.326.[2] Mean wind speed when the HVAC was off (WS_{off}) were 0.2, 0.18, 0.19, and 0.1 m s^{-1} in Rooms 132, 137, 140 and 171, respectively. Room 171 used central air handling. In Room 171 f_{on} was 0.675. Mean wind speed when the HVAC was on (WS_{on}) was 0.3 m s^{-1} in Room 171. The deployment time was t = 42 days for all rooms. K_{PUF} was averaged for coeluting congeners, and temperature was corrected according to Shoeib et al. 2002 and Herkert et al. 2016 (**Equations S3 – S5**).[3, 4] Previous studies indicate that airborne PCBs are almost completely in the gas phase, however, the PUF–PAS method captures both gas phase and fine particles. The V_{eff} applies to both phases.[3, 4] Concentrations from room air are reported in ng m^{-3}. We collected four 24-hour air samples using a low-volume sampler in Room 137 to confirm our concentration measurements via PUF-PAS. The sampler pulls air through a PUF plug at a rate of 2 L min^{-1}.[5]

The variable KPUF is calculated by the empirical equation (Shoeib[3]):

$$\log K_{PUF} = 0.6366 \log K_{OA} - 3.1774$$ **Equation S3**

where K_{OA} is calculated by (Herkert[4]):

$$\log K_{OA(T)} = \log K_{OA(25°C)} - \frac{\Delta U_{OA}}{2.303 \times R}\left(\frac{1}{T} - \frac{1}{298.15}\right)$$ **Equation S4**

where T is temperature (K), ΔUOA is the internal energy of octanol-air transfer (J mol-1), R is the gas constant (J mol^{-1} K^{-1}). The final concentration in air, C_{air}, is:

$$C_{air} = \frac{M_{PCB}}{V_{eff}}$$ **Equation S5**

where M_{PCB} is the concentration of a PCB (ng).

The accumulation of PCBs on PUF is dependent on the partitioning coefficient between PUF and air (K_{PUF}). K_{PUF} is dependent on the temperature-dependent octanol-air partitioning coefficient (**Equation S3**). A decrease in the effective sampling volume of a congener will lead to an increased mass of PCBs accumulating on PUF per unit time (**Equation S1**).

For PUF-PES, the PUF disk (same parameters as the PUF disk used in PUF-PAS) was placed at the bottom of a glass petri dish and secured with a thin strip of metal. The petri dish was then placed on top of a flat surface, with an air gap between the surface and the PUF. Gas-phase PCBs emitted from the surface adsorbed onto the PUF. PUF-PES were placed on the tile overlaid with carpet and wood panel in triplicate in Rooms 132 and 137. They were also placed in triplicate on

the hallway tile outside of Room 137. The PUF-PES were deployed for 23 days each (July 14, 2021, to August 6, 2021) in Room 132, Room 137, and the hallway. We repeated the deployment of new PUF-PES in triplicate in the same locations after a standard wipe test to evaluate emissions after removal of PCBs and other materials on the target surface. These 'post-wipe' PUF-PES were also deployed for 23 days (December 15, 2021, to January 7, 2022).

Gauze wipes were Fisherbrand nonsterile 2"x2" 12-ply 100% cotton gauze sponges. Gauze wipes did not undergo any cleaning prior to deployment. The glass vials and caps were rinsed with methanol, acetone, and hexane prior to wipe collection. We used 2 mL of hexane for each wipe for one minute each. We conducted standard wipe tests in the same locations the PUF-PES were deployed initially in triplicate in Rooms 132 and 137. The same was done for the hallway tile. After wiping, gauze was placed in 15 – 20 mL of hexane in individual glass vials. All standard gauze wipe tests were conducted on December 15, 2021. All samples were stored at −4 °C until extraction. All samples were extracted within 4 days of collection.

Bulk concentration of carpet (n = 6) and wall paneling (n = 6) were evaluated. The intra-room flooring was comprised of tile (standard 12-inch vinyl) with an overlay of multicolored carpet. The wall panel consisted of plywood and/or pressed fiberboard attached to cinder blocks with an adhesive. We collected samples of these materials using a standard industrial box cutter. We collected triplicate pieces of carpet and wood panel from Room 132 and 137. The carpet and wood panel were extracted from Rooms 132 and 137 on May 5, 2022.

We used accelerated solvent extraction with acetone and hexane (1:1 v/v) to extract PCBs from PUF samples, wipe samples, and bulk material samples. We used accelerated solvent extraction, turbulent evaporation with nitrogen gas, and acidified silica gel columns to extract and clean all PUF and gauze samples as detailed in Appendix B and previous studies.[6] Gas chromatography tandem mass spectrometry (Agilent 7000 Triple Quad with Agilent 7890A GC and Agilent 7693 autosampler) in multiple reaction monitoring mode (MRM) was used for identification and quantification of 209 PCBs as 171 chromatographic peaks. Information about the GC and MS instrument parameters is in Appendix B. We quantified PCBs using a calibration standard (AccuStandard, New Haven, CT) containing all 209 PCBs (25 ng mL^{-1} of mono- through trichlorinated congeners, 50 ng mL^{-1} tetra- through heptachlorinated congeners, and 75 ng mL^{-1} octa- through decachlorinated congeners) and the surrogate (d-PCB 65) and internal (d-PCB 30) standard. We used three hexane blanks before and after the calibration run and after sample runs to ensure no carryover. We identified PCBs by comparing samples with the same MRM transition according to retention time (±0.07 min) except where peak shape or surrogate standard shift dictated otherwise. Every PCB congener in every sample was assigned a peak area. Congeners with no discernable peaks were assigned an area of 1 such that there was a measurable limit of quantification.

Material Sample Parameters

Table S2. Weights and dimensions for each material sample collected on May 3, 2022. 'WP' is an abbreviation for wood panel and 'C' is an abbreviation for carpet.

	**Room 132 C01**	**Room 132 C02**	**Room 137 C01**	**Room 137 C02**	**Room 137 C03**	**Room 132 WP01**	**Room 132 WP02**	**Room 132 WP03**	**Room 137 WP01**	**Room 137 WP02**	**Room 137 WP03**
**Weight (g)**	1.37	1.26	1.13	1.30	1.18	2.26	3.77	4.46	3.32	3.27	2.89
**Surface Area (cm^2)**	4.94	4.84	3.63	4.84	4.84	10.08	13.31	13.61	12.75	13.31	11.09
**Height (cm)**	0.64	0.64	0.64	0.64	0.64	0.32	0.32	0.32	0.32	0.32	0.32
**Volume (cm^3)**	3.13	3.07	2.30	3.07	3.07	3.21	4.23	4.33	4.05	4.23	3.53

Instrument Parameters

The GC (Agilent 7000 Triple Quad with Agilent 7890A GC and Agilent 7693 autosampler) was equipped with a Supelco SPB-Octyl capillary column (Poly (50% n-octyl/50% methyl siloxane, 30 m × 0.25 mm ID, 0.25 μm film thicknesses)) with UHP helium as the carrier gas (2.25 mL/min) and UHP nitrogen as the collision gas (1.5 mL/min). The GC operated in solvent vent injection mode at the following injection conditions: initial temperature 45 °C, initial time 0.06 min, ramp 600 °C/min to inlet temperature 325 °C at 0.751 mL/min. The GC oven temperature program was 45 °C for 2 min, 45 to 75 °C at 100 °C/min and hold for 5 min, 75 to 150 °C at 15 °C/min and hold for 1 min, 150 to 280 at 2.5 °C/min and final hold 5 min (total run time 70.86 min). The MS transfer line temperature was held at 280 °C. The triple quadrupole MS electron ionization source was set to 260 °C. The MS-MS operated with the precursor-product transitions in **Table S3**.

Table S3. PCB precursor and product masses of unlabeled and deuterated calibration standards used in multiple reaction monitoring (MRM) mode on the triple quadrupole mass spectrometer[a]

Cl homolog	Precursor Mass	Product Mass
mono	188	152
di	222	152
tri	258	186
tetra	291.9	222
penta	325.9	255.9
hexa	359.8	289.9
hepta	393.8	323.9
octa	429.7	359.8
nona	463.7	393.8
deca	497.7	427.9
D5 tri	261	191
D5 tetra	296.9	277

[a]Unlabeled standards were from AccuStandard, New Haven, CT, USA. Labeled standards were from C/D/N Isotopes, Pointe-Claire, QC, Canada.

Limits of Quantification

Method blanks were used to monitor the laboratory background levels. Field blanks were used to monitor the background levels to and from the sampling site. We calculated the limit of quantification (LOQ) as the average congener mass in field and method blanks in the PUF-PAS (n = 4), PUF-PES before wipe (n = 5), PUF-PES after wipe (n = 3), gauze wipes (n = 6), and empty ASE cells used in material extractions (n = 9) plus two times the standard deviation. Congener masses below LOQ are reported as 0 because they are not significantly above the background levels observed in the laboratory or in transit from deployment to analysis and we cannot identify how much of the observed measurement is from the material or room sampled. LOQ values in PUF-PAS (**Table S4**) range between 2.75 x 10^{-4} and 6.21 ng per congener. The average sum of PCBs in the PUF-PAS method and field blanks is 14.71 ng. LOQ values in pre-wipe PUF-PES (**Table S5**) range between 0.02 and 1.41 ng per congener. The average sum of PCBs in the pre-wipe PUF-PES method and field blanks is 39.21 ng. LOQ values in post-wipe PUF-PES (**Table S6**) range between 5 x 10^{-3} and 1.01 ng per congener. The average sum of PCBs in the post-wipe PUF-PES method blanks is 9.13 ng. LOQ values in gauze wipes (**Table S7**) range between 0.04 and 0.83 ng per congener. The average sum of PCBs in the gauze wipe method and field blanks is 9.00 ng. LOQ values in PUF-PES deployed over aluminum foil (**Table S8**) range between 0.02 and 0.69 ng per congener. The average sum of PCBs in the method blanks from the extraction of PUF-PES deployed over aluminum foil is 0.16 ng. LOQ values in pieces of carpet (**Table S9**) range between 0.01 ng and 11.11 ng per congener. LOQ values in pieces of wood panel (**Table S10**) range between 0.02 ng and 5.80 ng per congener. The average sum of PCBs in the materials method blanks is 23.62 ng.

Table S4. Limit of quantification (LOQ) for each PCB congener or group of co-eluting congeners in units of nanograms from PUF-PAS measurements. *a*

PCB	LOQ	PCB	LOQ	PCB	LOQ	PCB	LOQ
1	0.16987	51	0.17173	106	0.09598	161	0.11272
2	0.01522	52	4.47691	107	0.08554	162	0.00027
3	0.33654	54	0.05518	108+124	0.05661	164	0.23603
4	0.62701	55	0.34358	110	3.71464	165	0.03002
5	0.18350	56	0.00086	111	0.19673	167	0.00032
6	0.40708	57	0.00085	112	0.06511	169	0.02766
7	0.12193	58	0.00077	114	0.42476	170	0.12477
8	2.23693	59+62+75	0.90025	115	6.20569	171+173	0.10093
9	0.13767	60	0.00081	117	2.31388	172	0.04715
10	0.16192	61+70+74+76	2.15208	118	0.77985	174	0.09382
11	3.34992	63	0.34231	120	0.10215	175	0.02335
12+13	2.42799	64	2.67490	121	0.04038	176	0.06419

15	1.85179	66	0.88645	122	0.27706	177	0.05824
16	2.17000	67	0.03403	123	0.16915	178	0.03415
17	2.24999	68	0.10621	126	0.01608	179	0.06133
18+30	4.36129	72	0.10140	127	0.07278	180+193	0.13994
19	0.45220	73	0.28582	129+138+163	1.68095	181	0.66175
20+28	2.22642	77	0.00112	130	0.21014	182	2.11347
21+33	5.30682	78	0.00104	131	0.12561	183	0.07156
22	2.85916	79	0.00098	132	0.57227	184	2.64543
23	0.05886	80	0.00075	133	0.11666	185	0.05042
24	0.14228	81	0.17533	134	0.13886	186	0.26141
25	0.27815	82	1.64733	135+151	0.25682	187	0.05126
26+29	0.76010	83	0.16387	136	0.18177	188	1.66828
27	0.62017	84	1.04023	137	0.27933	189	0.00044
31	2.64750	85+116	2.05296	139+140	0.39506	190	0.03522
32	1.58854	86+97+109+119	0.72239	141	0.37876	191	0.02273
34	0.00050	87+125	1.60094	142	0.08478	192	0.03381
35	0.00055	88	0.04800	143	0.20710	194	0.03427
36	0.00047	89	0.22626	144	0.09427	195	0.02777
37	0.00048	90+101+113	2.33606	145	0.08263	196	0.03068
38	0.00036	91	0.35607	146	0.12826	197	0.49681
39	0.00054	92	0.38338	147+149	0.90154	198+199	0.04866
40+71	1.91780	93+100	0.24972	148	0.28783	200	0.18736
41	0.59238	94	0.09142	150	0.21220	201	0.02095
42	1.86261	95	1.80843	152	0.06430	202	0.04279
43	1.02073	96	0.06319	153+168	0.88476	203	0.03032
44+47+65	4.35349	98	0.12845	154	0.32087	205	0.02965
45	1.94456	99	1.28571	155	0.14747	206	0.04594
46	0.69812	102	0.34385	156+157	0.00035	207	0.03071
48	1.41562	103	0.05588	158	0.37405	208	0.02183
49+69	2.40760	104	0.03825	159	0.00030	209	0.05218

50+53	1.00826	105	0.35985	160	0.20981		

[a] The LOQ was calculated as the upper limit of the 95% confidence interval of the mass in the blanks (average + 2*standard deviation).

Table B-1. Limit of quantification (LOQ) for each PCB congener or group of co-eluting congeners in units of nanograms for PUF-PES before hexane wiping. [a]

PCB	LOQ	PCB	LOQ	PCB	LOQ	PCB	LOQ
1	0.28297	51	0.11736	106	0.04461	161	0.02157
2	0.20746	52	1.40746	107	0.06297	162	0.01666
3	0.20164	54	0.10568	108+124	0.08494	164	0.02656
4	0.50130	55	0.07919	110	0.88831	165	0.02015
5	0.07226	56	0.15971	111	0.03407	167	0.02943
6	0.29272	57	0.03923	112	0.04092	169	0.01560
7	0.09359	58	0.05873	114	0.09703	170	0.12870
8	1.19210	59+62+75	0.18062	115	0.08743	171+173	0.04732
9	0.14633	60	0.11776	117	0.18411	172	0.05080
10	0.28838	61+70+74+76	1.23659	118	0.49428	174	0.04799
11	0.25768	63	0.10365	120	0.04747	175	0.05965
12+13	0.12940	64	0.30181	121	0.02455	176	0.02891
15	0.28666	66	0.37511	122	0.06749	177	0.07201
16	0.27094	67	0.07669	123	0.06692	178	0.18256
17	0.33259	68	0.09734	126	0.01602	179	0.02627
18+30	0.60834	72	0.07181	127	0.04281	180+193	0.09863
19	0.12609	73	0.06525	129+138+163	0.15548	181	0.05191
20+28	0.39722	77	0.07528	130	0.04168	182	0.07222
21+33	0.33225	78	0.09301	131	0.03445	183	0.04968
22	0.15516	79	0.06708	132	0.12265	184	0.05823
23	0.05395	80	0.04402	133	0.03507	185	0.02993
24	0.05344	81	0.07382	134	0.06468	186	0.02421
25	0.08281	82	0.13192	135+151	0.11208	187	0.05655
26+29	0.14730	83	0.06100	136	0.07962	188	0.04077
27	0.07954	84	0.53274	137	0.04566	189	0.14266

31	0.38760	85+116	0.19242	139+140	0.05681	190	0.14551
32	0.17294	86+97+109+119	0.44093	141	0.05686	191	0.07304
34	0.05418	87+125	0.48026	142	0.02882	192	0.04175
35	0.04853	88	0.03382	143	0.01857	194	0.11235
36	0.04183	89	0.06999	144	0.05298	195	0.07181
37	0.10920	90+101+113	1.27316	145	0.02265	196	0.07497
38	0.04820	91	0.17994	146	0.04914	197	0.04423
39	0.04239	92	0.31494	147+149	0.23312	198+199	0.07888
40+71	0.22495	93+100	0.07839	148	0.02108	200	0.04370
41	0.11107	94	0.05721	150	0.02535	201	0.02818
42	0.15678	95	1.27274	152	0.02105	202	0.02499
43	0.07733	96	0.06293	153+168	0.17131	203	0.05819
44+47+65	0.72385	98	0.01877	154	0.03451	205	0.09962
45	0.12461	99	0.47058	155	0.03007	206	0.18124
46	0.08947	102	0.12518	156+157	0.02030	207	0.05192
48	0.11915	103	0.06477	158	0.03621	208	0.03856
49+69	0.44373	104	0.06431	159	0.02316	209	0.14300
50+53	0.22012	105	0.14207	160	0.01864		

[a] The LOQ was calculated as the upper limit of the 95% confidence interval of the mass in the blanks (n = 5) (average + 2*standard deviation).

Table S6. Limit of quantification (LOQ) for each PCB congener or group of co-eluting congeners in units of nanograms for PUF-PES after hexane wiping. [a]

PCB	LOQ	PCB	LOQ	PCB	LOQ	PCB	LOQ
1	0.05009	51	0.03335	106	0.01768	161	0.01876
2	0.04351	52	0.40478	107	0.05486	162	0.02644
3	0.03958	54	0.05430	108+124	0.03698	164	0.03652
4	0.07341	55	0.02175	110	0.85939	165	0.01515
5	0.06336	56	0.08886	111	0.01766	167	0.03409
6	0.06995	57	0.02994	112	0.00753	169	0.02885
7	0.05141	58	0.02874	114	0.11716	170	0.08600
8	0.16741	59+62+75	0.06476	115	0.17057	171+173	0.03041

9	0.05257	60	0.05883	117	0.15993	172	0.03806
10	0.04644	61+70+74+76	0.48451	118	0.60197	174	0.13943
11	0.15267	63	0.04076	120	0.01755	175	0.01819
12+13	0.05851	64	0.10626	121	0.01875	176	0.02188
15	0.04542	66	0.14946	122	0.02229	177	0.07216
16	0.06395	67	0.03223	123	0.01726	178	0.04180
17	0.06290	68	0.06369	126	0.02352	179	0.06833
18+30	0.11107	72	0.03087	127	0.02310	180+193	0.23625
19	0.05496	73	0.03306	129+138+163	0.65905	181	0.10488
20+28	0.09779	77	0.07402	130	0.04390	182	0.18582
21+33	0.07797	78	0.04160	131	0.02851	183	0.10015
22	0.04560	79	0.04362	132	0.23098	184	0.18088
23	0.02398	80	0.03617	133	0.02859	185	0.02064
24	0.02488	81	0.05212	134	0.07505	186	0.03943
25	0.03068	82	0.10009	135+151	0.25710	187	0.16164
26+29	0.04462	83	0.03539	136	0.11198	188	0.12338
27	0.02469	84	0.30079	137	0.04235	189	0.03733
31	0.09372	85+116	0.13112	139+140	0.03715	190	0.03608
32	0.05091	86+97+109+119	0.29950	141	0.10005	191	0.03239
34	0.01627	87+125	0.35798	142	0.01860	192	0.01689
35	0.02141	88	0.12929	143	0.02485	194	0.11440
36	0.01197	89	0.01936	144	0.03045	195	0.04263
37	0.03386	90+101+113	1.01378	145	0.01516	196	0.09123
38	0.01902	91	0.02655	146	0.08515	197	0.03453
39	0.01838	92	0.21877	147+149	0.60143	198+199	0.15029
40+71	0.10330	93+100	0.02923	148	0.02008	200	0.03800
41	0.03879	94	0.02183	150	0.01638	201	0.02561
42	0.08841	95	0.75235	152	0.01187	202	0.05253
43	0.03052	96	0.01861	153+168	0.44327	203	0.10151
44+47+65	0.23471	98	0.00517	154	0.02283	205	0.02824

45	0.07058	99	0.32569	155	0.01431	206	0.10823
46	0.04192	102	0.03366	156+157	0.07243	207	0.04339
48	0.04078	103	0.01991	158	0.07760	208	0.04097
49+69	0.14872	104	0.02046	159	0.02121	209	0.06275
50+53	0.10554	105	0.21442	160	0.01043		

[a] The LOQ was calculated as the upper limit of the 95% confidence interval of the mass in the blanks (n = 3) (average + 2*standard deviation).

Table S7. Limit of quantification (LOQ) for each PCB congener or group of co-eluting congeners in units of nanograms for gauze wipes with hexane. [a]

PCB	LOQ	PCB	LOQ	PCB	LOQ	PCB	LOQ
1	0.05783	51	0.09713	106	0.12295	161	0.12324
2	0.04112	52	0.47507	107	0.11835	162	0.10231
3	0.08639	54	0.07954	108+124	0.17703	164	0.08110
4	0.18758	55	0.16604	110	0.44259	165	0.11973
5	0.04209	56	0.14796	111	0.14378	167	0.09260
6	0.07798	57	0.10640	112	0.08215	169	0.15860
7	0.04083	58	0.10318	114	0.19809	170	0.27726
8	0.26460	59+62+75	0.22031	115	0.19599	171+173	0.29588
9	0.03796	60	0.14454	117	0.26305	172	0.27597
10	0.03638	61+70+74+76	0.65915	118	0.53522	174	0.12408
11	0.32906	63	0.08506	120	0.08894	175	0.09625
12+13	0.20244	64	0.20043	121	0.10083	176	0.17992
15	0.08297	66	0.26154	122	0.14986	177	0.12250
16	0.18762	67	0.17495	123	0.13430	178	0.08745
17	0.15964	68	0.14359	126	0.06014	179	0.19763
18+30	0.22900	72	0.09168	127	0.09150	180+193	0.19649
19	0.09033	73	0.11091	129+138+163	0.39421	181	0.15750
20+28	0.28114	77	0.08496	130	0.12347	182	0.13723
21+33	0.25030	78	0.04043	131	0.09178	183	0.15285
22	0.14512	79	0.09833	132	0.16991	184	0.13115
23	0.05443	80	0.11517	133	0.10904	185	0.08427

24	0.04694	81	0.13682	134	0.16629	186	0.13801
25	0.07048	82	0.33843	135+151	0.17944	187	0.12107
26+29	0.10984	83	0.16920	136	0.10255	188	0.12386
27	0.05167	84	0.35953	137	0.07749	189	0.12003
31	0.22830	85+116	0.18970	139+140	0.13420	190	0.12749
32	0.09800	86+97+109+119	0.31731	141	0.12657	191	0.10294
34	0.05167	87+125	0.44769	142	0.12913	192	0.19479
35	0.05699	88	0.14653	143	0.07262	194	0.16986
36	0.06309	89	0.25812	144	0.09425	195	0.12892
37	0.09293	90+101+113	0.83363	145	0.07677	196	0.19326
38	0.05837	91	0.21709	146	0.09254	197	0.28253
39	0.05953	92	0.26194	147+149	0.30325	198+199	0.15059
40+71	0.29935	93+100	0.11862	148	0.07638	200	0.11552
41	0.17446	94	0.17035	150	0.07182	201	0.14080
42	0.20600	95	0.63443	152	0.06629	202	0.13913
43	0.10287	96	0.08740	153+168	0.25179	203	0.10213
44+47+65	0.49834	98	0.15769	154	0.05713	205	0.22638
45	0.17938	99	0.31583	155	0.05546	206	0.22802
46	0.17596	102	0.16072	156+157	0.15883	207	0.14312
48	0.21647	103	0.14466	158	0.10312	208	0.15463
49+69	0.31448	104	0.07311	159	0.10731	209	0.06275
50+53	0.19642	105	0.38316	160	0.08361		

[a] The LOQ was calculated as the upper limit of the 95% confidence interval of the mass in the blanks (n = 6) (average + 2*standard deviation).

Table S8. Limit of quantification (LOQ) for each PCB congener or group of co-eluting congeners in units of nanograms for PUF-PES over aluminum foil. [a]

PCB	LOQ	PCB	LOQ	PCB	LOQ	PCB	LOQ
1	0.05490	51	0.11530	106	0.16020	161	0.06423
2	0.06771	52	0.38010	107	0.13085	162	0.05985
3	0.07978	54	0.13886	108+124	0.29853	164	0.06821
4	0.06184	55	0.26742	110	0.39443	165	0.09854

5	0.05431	56	0.30315	111	0.12547	167	0.07888
6	0.08081	57	0.16049	112	0.19732	169	0.02607
7	0.06262	58	0.09820	114	0.19493	170	0.12338
8	0.11534	59+62+75	0.35080	115	0.40487	171+173	0.10766
9	0.06409	60	0.14278	117	0.24146	172	0.10425
10	0.04290	61+70+74+76	0.68715	118	0.21394	174	0.11628
11	0.26146	63	0.14928	120	0.11647	175	0.08595
12+13	0.15071	64	0.22880	121	0.11203	176	0.10646
15	0.10778	66	0.28700	122	0.17063	177	0.06099
16	0.11385	67	0.18932	123	0.12730	178	0.14964
17	0.11008	68	0.19487	126	0.06867	179	0.10673
18+30	0.14211	72	0.12600	127	0.09683	180+193	0.09277
19	0.05760	73	0.15471	129+138+163	0.26966	181	0.08941
20+28	0.18615	77	0.15722	130	0.10746	182	0.18767
21+33	0.15969	78	0.14076	131	0.09745	183	0.06935
22	0.11904	79	0.14525	132	0.13576	184	0.24758
23	0.06203	80	0.15591	133	0.11069	185	0.07528
24	0.04863	81	0.14264	134	0.25164	186	0.09304
25	0.12413	82	0.28127	135+151	0.29302	187	0.10673
26+29	0.15271	83	0.29965	136	0.13455	188	0.14217
27	0.06914	84	0.39492	137	0.11024	189	0.05243
31	0.15220	85+116	0.26479	139+140	0.16272	190	0.07629
32	0.08993	86+97+109+119	0.35185	141	0.12357	191	0.06232
34	0.08946	87+125	0.66464	142	0.10909	192	0.05092
35	0.07110	88	0.17228	143	0.08192	194	0.06405
36	0.10435	89	0.17818	144	0.11263	195	0.07605
37	0.11594	90+101+113	0.68171	145	0.10593	196	0.06748
38	0.07564	91	0.14947	146	0.07468	197	0.09109
39	0.12609	92	0.20420	147+149	0.26720	198+199	0.09590
40+71	0.45437	93+100	0.29988	148	0.07576	200	0.08697

41	0.26975	94	0.18201	150	0.11156	201	0.08200
42	0.25156	95	0.46547	152	0.11750	202	0.09101
43	0.32976	96	0.15670	153+168	0.14874	203	0.05294
44+47+65	0.54106	98	0.22467	154	0.09245	205	0.05037
45	0.24996	99	0.30615	155	0.08623	206	0.08190
46	0.23727	102	0.27780	156+157	0.09131	207	0.07652
48	0.25805	103	0.19001	158	0.07749	208	0.07693
49+69	0.30489	104	0.11455	159	0.06630	209	0.09815
50+53	0.35081	105	0.18653	160	0.08697		

[a] The LOQ was calculated as the upper limit of the 95% confidence interval of the mass in the blanks (n = 3) (average + 2*standard deviation).

Table S9. Limit of quantification (LOQ) for each PCB congener or group of co-eluting congeners in units of nanograms for carpet material. [a]

PCB	LOQ	PCB	LOQ	PCB	LOQ	PCB	LOQ
1	0.141488	51	0.121118	106	0.037048	161	0.031028
2	0.134117	52	1.176336	107	0.784168	162	0.044996
3	0.152203	54	0.080879	108+124	0.527836	164	0.377689
4	0.146385	55	0.092745	110	8.158791	165	0.036821
5	0.097484	56	1.07165	111	0.055992	167	0.423936
6	0.113555	57	0.096372	112	0.068609	169	0.01427
7	0.11977	58	0.087904	114	0.630731	170	0.291881
8	0.17012	59+62+75	0.187789	115	0.110991	171+173	0.139137
9	0.126806	60	0.529024	117	0.303792	172	0.110909
10	0.123245	61+70+74+76	6.025804	118	11.11257	174	0.169165
11	0.174423	63	0.10076	120	0.071843	175	0.123109
12+13	0.14717	64	0.381787	121	0.050538	176	0.070172
15	0.082046	66	2.100005	122	0.293465	177	0.132712
16	0.065749	67	0.085388	123	0.290785	178	0.056264
17	0.073799	68	0.099167	126	0.919942	179	0.114609
18+30	0.200967	72	0.068661	127	0.092036	180+193	0.625402
19	0.077761	73	0.077395	129+138+163	5.700111	181	0.062298

20+28	0.115692	77	0.149578	130	0.33773	182	0.043944
21+33	0.136791	78	0.089086	131	0.086316	183	0.137533
22	0.059358	79	0.078268	132	0.831013	184	0.060049
23	0.062617	80	0.049396	133	0.119282	185	0.031632
24	0.075741	81	0.074431	134	0.184717	186	0.061431
25	0.071407	82	2.107055	135+151	0.344642	187	0.088676
26+29	0.117083	83	0.218285	136	0.185574	188	0.049349
27	0.055242	84	1.049885	137	1.051959	189	0.062864
31	0.103661	85+116	2.021495	139+140	0.118656	190	0.068683
32	0.052753	86+97+109+119	2.433944	141	0.593558	191	0.115221
34	0.050186	87+125	2.092601	142	0.022636	192	0.090267
35	0.033602	88	0.033711	143	0.041288	194	0.150304
36	0.043433	89	0.091992	144	0.109432	195	0.102421
37	0.06835	90+101+113	4.489804	145	0.080857	196	0.056886
38	0.036899	91	0.490985	146	0.368683	197	0.067797
39	0.047029	92	0.574804	147+149	1.287388	198+199	0.107229
40+71	0.234526	93+100	0.13756	148	0.118168	200	0.090848
41	0.11078	94	0.076243	150	0.04126	201	0.031858
42	0.143922	95	1.748285	152	0.043026	202	0.037047
43	0.090812	96	0.050119	153+168	3.825488	203	0.054315
44+47+65	0.719401	98	0.081707	154	0.040495	205	0.100695
45	0.10488	99	3.206992	155	0.055752	206	0.093792
46	0.114929	102	0.140271	156+157	0.094716	207	0.097724
48	0.106661	103	0.062044	158	0.602548	208	0.104906
49+69	0.401252	104	0.072192	159	0.054241	209	0.090516
50+53	0.220425	105	7.573645	160	0.046023		

[a] The LOQ was calculated as the upper limit of the 95% confidence interval of the mass in the blanks (n = 3) (average + 2*standard deviation).

Table S10. Limit of quantification (LOQ) for each PCB congener or group of co-eluting congeners in units of nanograms for wood panel material. [a]

PCB	LOQ	PCB	LOQ	PCB	LOQ	PCB	LOQ

1	0.103695	51	0.136623	106	0.067472	161	0.039784
2	0.109537	52	0.725897	107	0.097259	162	0.029679
3	0.131887	54	0.095518	108+124	0.093071	164	0.059925
4	0.126067	55	0.135478	110	0.790054	165	0.039992
5	0.091691	56	0.146868	111	0.079046	167	0.02817
6	0.097012	57	0.102189	112	0.041787	169	0.046186
7	0.090149	58	0.097758	114	0.078783	170	0.089993
8	0.196906	59+62+75	0.202854	115	0.04446	171+173	0.08453
9	0.097887	60	0.137222	117	0.172288	172	0.05507
10	0.112837	61+70+74+76	0.732036	118	0.401921	174	0.063727
11	0.230737	63	0.117573	120	0.051837	175	0.057596
12+13	0.172812	64	0.139788	121	0.049304	176	0.052661
15	0.086603	66	0.151655	122	0.079479	177	0.067325
16	0.117611	67	0.094871	123	0.051992	178	0.062184
17	0.096855	68	0.129805	126	0.030061	179	0.123603
18+30	0.201339	72	0.084075	127	0.09029	180+193	0.172234
19	0.060702	73	0.074954	129+138+163	0.30226	181	0.053257
20+28	0.197571	77	0.102876	130	0.0423	182	0.07195
21+33	0.166158	78	0.102377	131	0.05469	183	0.044944
22	0.072979	79	0.09536	132	0.104584	184	0.075464
23	0.040346	80	0.103531	133	0.068729	185	0.03756
24	0.068815	81	0.070741	134	0.056343	186	0.095461
25	0.053983	82	0.160452	135+151	0.196695	187	0.08462
26+29	0.105465	83	0.165813	136	0.105398	188	0.046513
27	0.05835	84	0.263688	137	0.137468	189	0.130285
31	0.15011	85+116	0.21542	139+140	0.078284	190	0.076092
32	0.074971	86+97+109+119	0.484862	141	0.069851	191	0.041013
34	0.052048	87+125	0.408466	142	0.069053	192	0.102951
35	0.045997	88	0.045222	143	0.060949	194	0.153701
36	0.037359	89	0.079545	144	0.063671	195	0.116551

37	0.056616	90+101+113	0.903674	145	0.08846	196	0.12949
38	0.049866	91	0.1818	146	0.07633	197	0.034989
39	0.054876	92	0.157252	147+149	0.334563	198+199	0.186126
40+71	0.209154	93+100	0.137775	148	0.038633	200	0.052795
41	0.108221	94	0.085409	150	0.053405	201	0.051244
42	0.109136	95	0.883774	152	0.043961	202	0.053144
43	0.081095	96	0.059033	153+168	0.335704	203	0.132786
44+47+65	0.550057	98	0.082744	154	0.043148	205	0.126418
45	0.104617	99	0.35415	155	0.039435	206	0.179996
46	0.129356	102	0.076813	156+157	0.065254	207	0.073167
48	0.095572	103	0.067761	158	0.045498	208	0.117825
49+69	0.24909	104	0.04039	159	0.051623	209	0.102412
50+53	0.17283	105	0.204239	160	0.04455		

[a] The LOQ was calculated as the upper limit of the 95% confidence interval of the mass in the blanks (n = 3) (average + 2*standard deviation).

Quality Control and Assurance

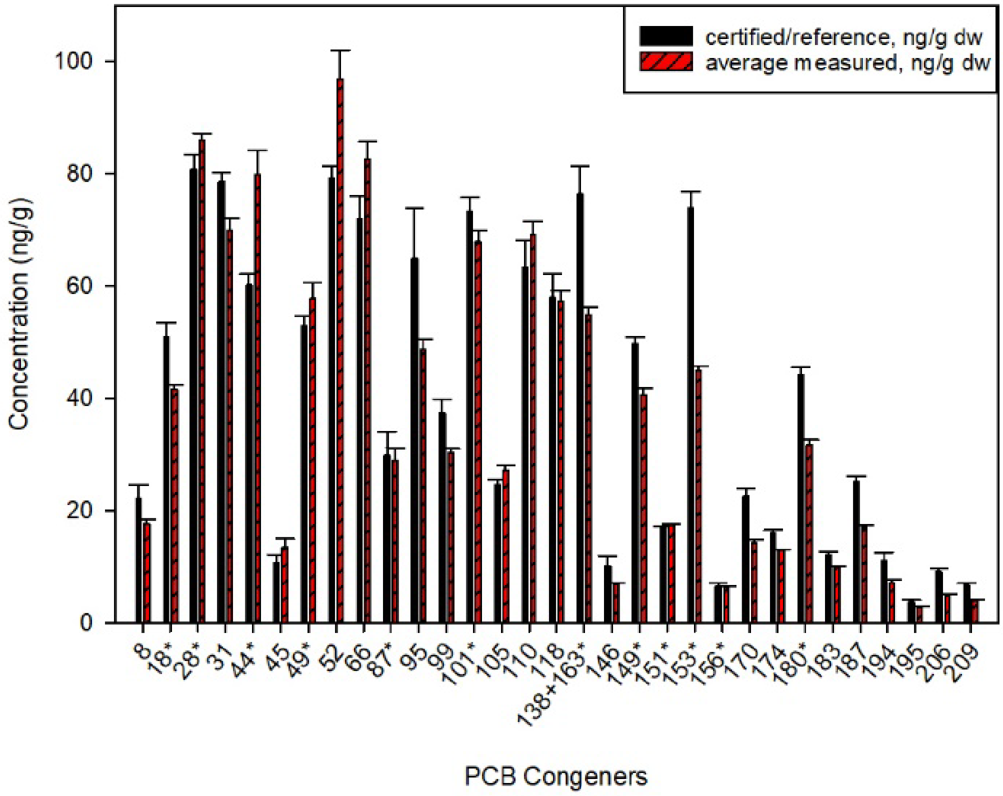

Figure S1. National Institute of Standards and Technology certified/reference concentrations compared to average measured concentrations (n=3) in standard reference material 1944 (New York/New Jersey Waterway Sediment, NIST Standard Reference Material 2585, Gaithersburg, MD, US). Error bars represent standard deviation (measured). The standard deviation of the NIST measurements were provided by the certificate. Measured concentrations are corrected for surrogate standard recovery. * Indicates difference between NIST certified/reference and measured values likely due to contribution from co-eluting congener(s) in the measured value. PCB congeners 138 and 163 are certified by NIST separately but were added together here because they co-elute in our method.

We corrected masses in samples where surrogate recoveries were less than 100%. Samples with surrogate recoveries above 100% were not corrected. The results from three PUF-PAS samples and one PUF-PES sample were not reported because the surrogate recoveries were outside our acceptable range of 50 – 110%. Average ± standard deviation of surrogate recoveries in PUF-PAS for PCB 14, d-PCB 65, and PCB 166 were 70.1% ± 19%, 90.5% ± 8%, and 96.7% ± 6%, respectively (**Figure S3**). Average ± standard deviation of surrogate recoveries in pre-wipe PUF-PES for PCB 14, d-PCB 65, and PCB 166 were 85.3% ± 11%, 94.1% ± 9%, and 95.2% ± 9%, respectively. Average ± standard deviation of surrogate recoveries in post-wipe PUF-PES for PCB 14, d-PCB 65, and PCB 166 were 87.0% ± 4%, 88.9% ± 8%, and 99.6% ± 1%, respectively. No congeners were corrected for the carpet bulk samples. Due to the coelution of PCB 128, which is prominent in Aroclor sources, with standard PCB 166 we did not correct the octa- through decachlorinated congeners for half of the wood panel samples. Average ± standard deviation of surrogate recoveries in pieces of wood panel for PCB 14, d-PCB 65, and PCB 166 were 66.3% ± 5%, 79.3% ± 8%, and 79.8% ± 10% respectively.

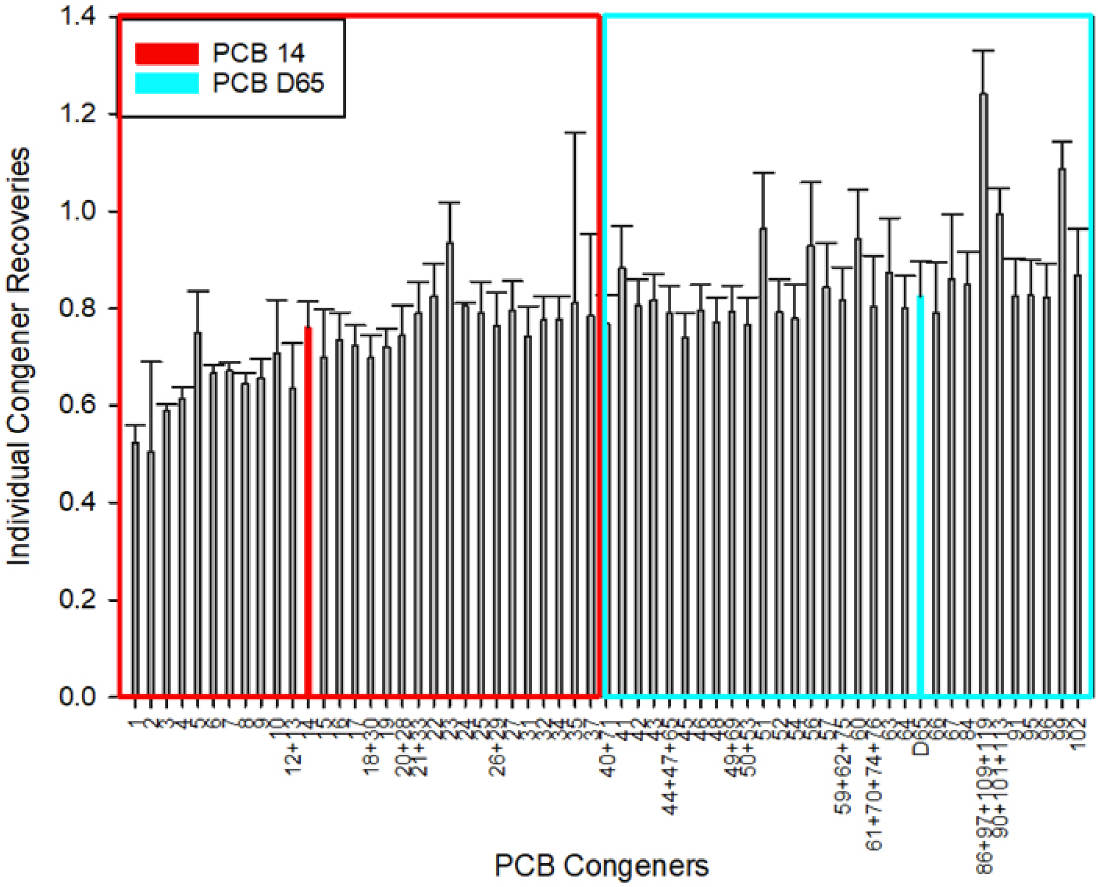

Figure S2. Recoveries from Aroclor 1016 spiked onto PUF (n = 3) (calculated as mass of congener in the sample divided by mass of congener in the reference). PCBs 103 – 209 are not depicted because they represent only 0.06% of the total mass. PCB 14 and d-PCB 65 are highlighted in red and blue, respectively. These results show that PCB 14 and d-PCB 65 are representative of the recoveries of lower- and middle-chlorinated PCBs and were used as surrogate standards to correct for mass lost during extraction for all samples in this study except field and method blanks. Three PUF disks were cleaned with 1:1 acetone/hexane solution and wrapped in aluminum foil. Prior to extraction, the PUF were spiked with 12,205 ng of Aroclor 1016 each (5 µL of 2441 µg mL^{-1} solution) using a 10 µL syringe. The same amount of Aroclor 1016 was spiked into a reference glass autosampler vial containing 0.7 µL of hexane.

We spiked and extracted three PUF with 12,205 ng of Aroclor 1016 to assess the recoveries of those PCBs compared to surrogate standards PCB 14 and d-PCB 65 and the appropriateness of using those surrogate standards to correct congener masses in samples (**Figure S2**). We used a large mass of Aroclor 1016 for this extraction to ensure congener specific masses were well above the LOQ resulting in clearer evaluation. Aroclor 1016 was chosen because it is comprised of 99.4% lower homolog groups which emphasizes the congeners typically corrected by PCBs

14 and d-65. The same three PUF and the reference were also spiked with 50.8 ng of PCB 14 and 52.5 ng of d-PCB 65 each. The data shown in Figure 3-1 confirms it is appropriate to correct PCBs 1-39 using recoveries of surrogate standard PCB 14. We have corrected PCB 1 – 39 accordingly throughout this study.

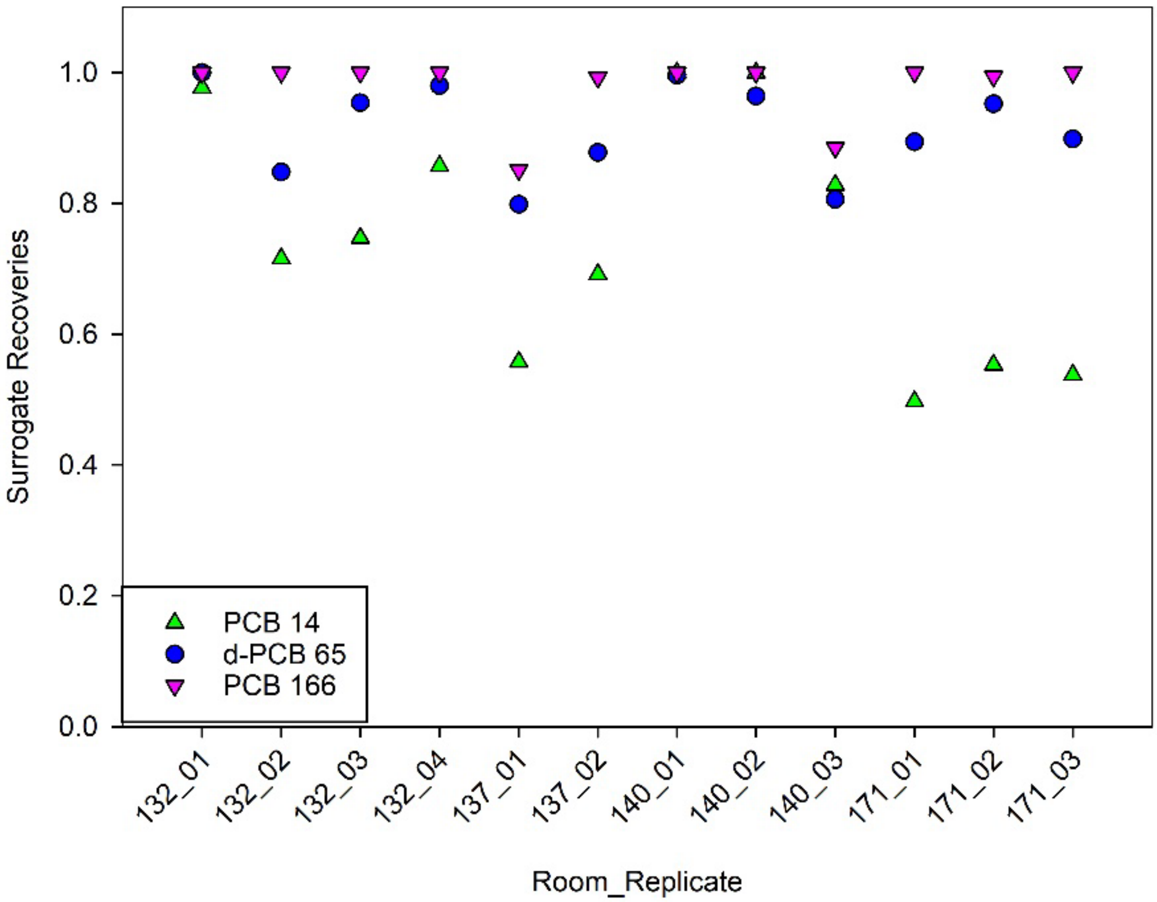

Figure S3. Recoveries of PCBs 14, d-65 and 166 from PUF-PAS in Rooms 132, 137, 140, and 171. Representativeness of extraction efficiency was assessed with these replicates. Data points of the same color and shape represent the same surrogate standard used. Low recoveries of PCB 14 can be caused due to less chlorination and high vaporization.[2] An extraction of Aroclor 1016 from PUF was performed to verify our ability to quantify PCB 14 and correct the proper congeners by this surrogate (**Figure S2**).

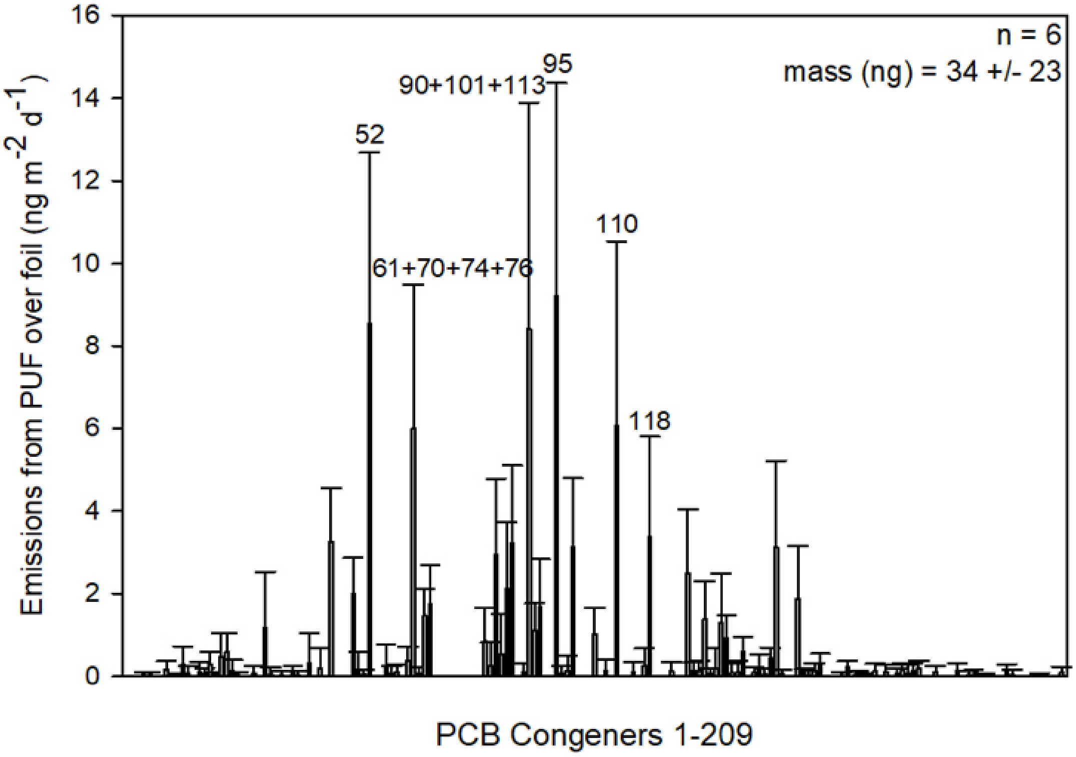

Figure S4. Emissions from all 209 PCB congeners from aluminum foil placed on a shelf in Room 171. The most significant congeners are labeled. Total emissions from this negative control method averaged 89 ± 65 ng m^{-2} d^{-1}. The lowest room material emission sample measured in our study was 1,305 ng m^{-2} d^{-1}.

To assure that the PUF-PES method captured emissions and was not significantly affected by leakage from room air, we created a negative control by deploying PUF-PES (n = 6) over a sheet of aluminum foil in Room 171 for 25 days (November 2 – 27, 2021) (**Figure S4**). Aluminum foil is known not to contain any PCBs. The average total PCBs collected on six PUF-PES replicates deployed over aluminum foil in Room 171 was 88.65 ± 65 ng m^{-2} d^{-1} (mass = 33.90 ± 23 ng). For comparison, the lowest emissions we measured was from post-wipe wood panel (n = 3) in Room 137. This measurement was 1413 ± 95 ng m^{-2} d^{-1} (mass = 828.55 ± 50 ng), indicating that leakage from room air accounts for less than 7% of the PCBs emitted from the surfaces measured in this study.

Statistical Tests

Wilcoxon Signed Rank Test: We calculated the statistical significance of the differences in congener-specific concentration before and after wiping the carpet laid on top of tile, wood panel walling, and hallway tile in each room with hexane using a Wilcoxon Signed Rank Test (**Equation S6**).[7] N_r is the sample size excluding pairs where x_1 is equal to x_2. The corresponding ranked pairs from two distributions are $x_{1,i}$ and $x_{2,i}$. R_i represents the rank.

$$W = \sum_{i=1}^{N_r} \left[sgn\left(x_{2,i} - x_{1,i}\right) * R_i \right] \qquad \textbf{Equation S6}$$

The congener values were not normalized for this non-parametric test of match-paired data because it would eliminate critical magnitude differences the test aims to evaluate. After conducting the Wilcoxon test, we used the Bonferroni method for correcting multiple sample testing. Statistical significance is defined by a 95% confidence interval ($\alpha \leq 0.05$).[8] The Bonferroni Correction states α must be divided by the number of tests (171 for each PCB congener and congener coelution evaluated) creating a new p-value that defines statistical significance: 2.92×10^{-4}.[9]

Cosine Theta Analysis: We evaluated the differences in congener profiles using cosine theta (cos θ).[1] Cos θ varies from 0 (no correlation) to 1 (complete correlation). We normalized the congener concentrations to the sum of the PCB congeners in each sample to evaluate the composition of the profiles.[6] Because all airborne PCBs are commonly assumed to originate from volatilization of Aroclors, we compared our measurements to the vapor-pressure normalized Aroclor profiles: we multiplied each congener fraction in each Aroclor mixture by congener-specific vapor pressures to yield a vaporized Aroclor profile. Airborne PCB congener distributions were compared to vaporized Aroclor profiles for all relevant cos θ analyses.[6, 10]

Linear Mixed-Effects Model: A mixed effects model was used to investigate statistical significance in instances where the numbers are not independent of one another (replicates from a single room) but there is more than one factor (multiple rooms and materials). It is an extension of a general linear regression. In this study we used a mixed effects model to identify the significant difference between emissions from tile with carpet overlay and wood panel walling before and after hexane wiping ($p = 6.92 \times 10^{-4}$ and 6.35×10^{-5} respectively). We also used a mixed effects model to determine that the bulk concentration of carpet is significantly higher than that of wood panel ($p = 4.06 \times 10^{-8}$). Lastly, a mixed effects model revealed there was no significant difference between the mass of PCBs wiped off intra-room materials ($p = 0.22$).

Random Effect Model: A random effects model is used when variables are constant over time and not correlated with independent variables. We used a random effects model to determine there were no significant differences in air concentration from room to room ($p = 0.80$).

Results and Discussion

Variability in Room-to-Room Air

We assessed whether this variability in concentration is typical of indoor air sampler replicates analyzed for all 209 PCBs deployed simultaneously with uncontrolled windspeeds by comparing our results to those of Herkert et al.'s 2018 studies (**Table S11**).[2, 11] This comparison shows that regardless of sample size (n) indoor air measurements of all 209 congeners from the same location over the same time period can be highly variable due to air speed differences in different parts of the room. This variability is reduced when using low-volume samplers as opposed to PUF-PAS. We used PUF-PAS here for precision in congener distribution which result in a long-term integrated average of total PCB concentration. Our PUF-PAS and low-volume sampler measurements had similar relative standard deviations further confirming the PUF-PAS measurements are representative of their environment. Throughout this study we observed that samples from the same room air had similar congener profiles even if concentrations differed

from one sampler to another. Nevertheless, we did not perform additional passive sampling or emission sampling in Room 171.

Table S11. PCB concentrations (ng m^{-3}) in Rooms 132, 137, 140, 171 and two rooms from Herkert et al.'s previous studies taken using PUF-PAS and low-volume samplers.

	**Indoor Air Sampler**	**Average Concentration (ng m^{-3})**	**Standard Deviation (±)**	**Relative Standard Deviation % (±)**	**n**	**Was Windspeed Measured?**
Room 132	PUF-PAS	103	25	25	4	Yes
Room 137	PUF-PAS	111	9	8	2	Yes
Room 137	Low-Volume	164	16	10	4	Yes
Room 140	PUF-PAS	53	20	38	3	Yes
Room 171	PUF-PAS	82	57	69	3	Yes
Herkert et al. 2018 (a)[11]	PUF-PAS	2	1	52	2	No
Herkert et al. 2018 (b)[2]	Low-Volume	8	2	22	4	Yes

Surface PCBs (Wipes)

We found removable PCBs on the surface of carpet, wood panel, and tile (**Table S12**). Wipe samples were conducted on the same location where initial emissions measurements had been taken. The surface area of each wiped location is the same as that of one PUF disk, 0.0153 m^2. The mass of PCBs on each wipe ranged from 176 ng (Room 137 carpet) to 1,324 ng (hallway tile). We calculated the total PCB mass associated with each surface in each room. Total masses wiped for wood panel averaged 2.0 x 10^5 ng in Room 132 and 4.2 x 10^5 ng in Room 137. In both rooms, 10x the mass of PCBs emitted in one day was instantaneously wiped off the wood panel. All surface wipe measurements regardless of material or location ranged between 10^4 – 10^5 ng m^{-2}, suggesting an even distribution of PCBs across the removable surface layer on different materials throughout the rooms. There has not been a significant reduction in wiped PCBs in this building from 2016 (7.9 x 10^4 ng m^{-2}) to 2022. We performed a mixed effects model that confirmed there is no significant difference between the masses wiped off carpet and wood panel regardless of location ($p = 0.22$). This evidence supports the existence of an evenly distributed removable surface PCB layer throughout all rooms. We did not compare the bare tile to intra-room materials due to its lack of localized environment within the building. However, bare tile surface PCB data can be found in the complete, published, dataset.

Table S12. Average and standard deviation of surface PCBs on wipe replicates and the corresponding average amount of PCBs for the entire surface area of the wood panel or carpet in Rooms 132 and 137.

**Wipe**<br>**Wood Panel (WP)**<br>**Carpet (C)**	**Average surface PCBs Measured (ng m^{-2})**	**Surface Area of Material in Room (m^2)**	**Average total Mass on Surface (ng)**	**n**
WP Room 132	2.60 ± 0.2 x 10^4	7.8	2.03 x 10^5	2
WP Room 137	5.42 ± 0.3 x 10^4	7.7	4.17 x 10^5	3
C Room 132	2.66 ± 1.6 x 10^4	20	5.32 x 10^5	3
C Room 137	3.07 ± 1.8 x 10^4	19.5	6.01 x 10^5	3

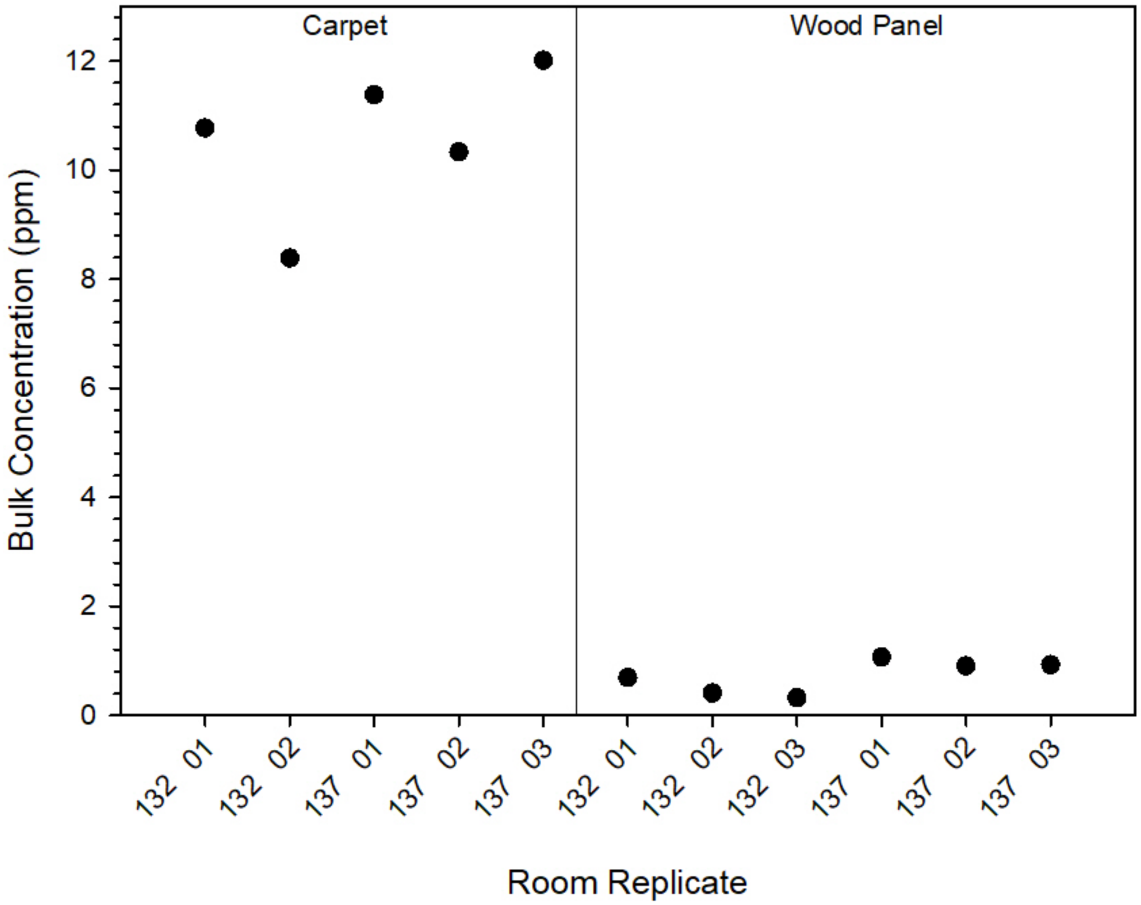

Figure S5. Material bulk PCB concentrations (parts per million) of from carpet and wood panel in Rooms 132 and 137. Samples were taken after hexane wiping.

Congener Similarities:

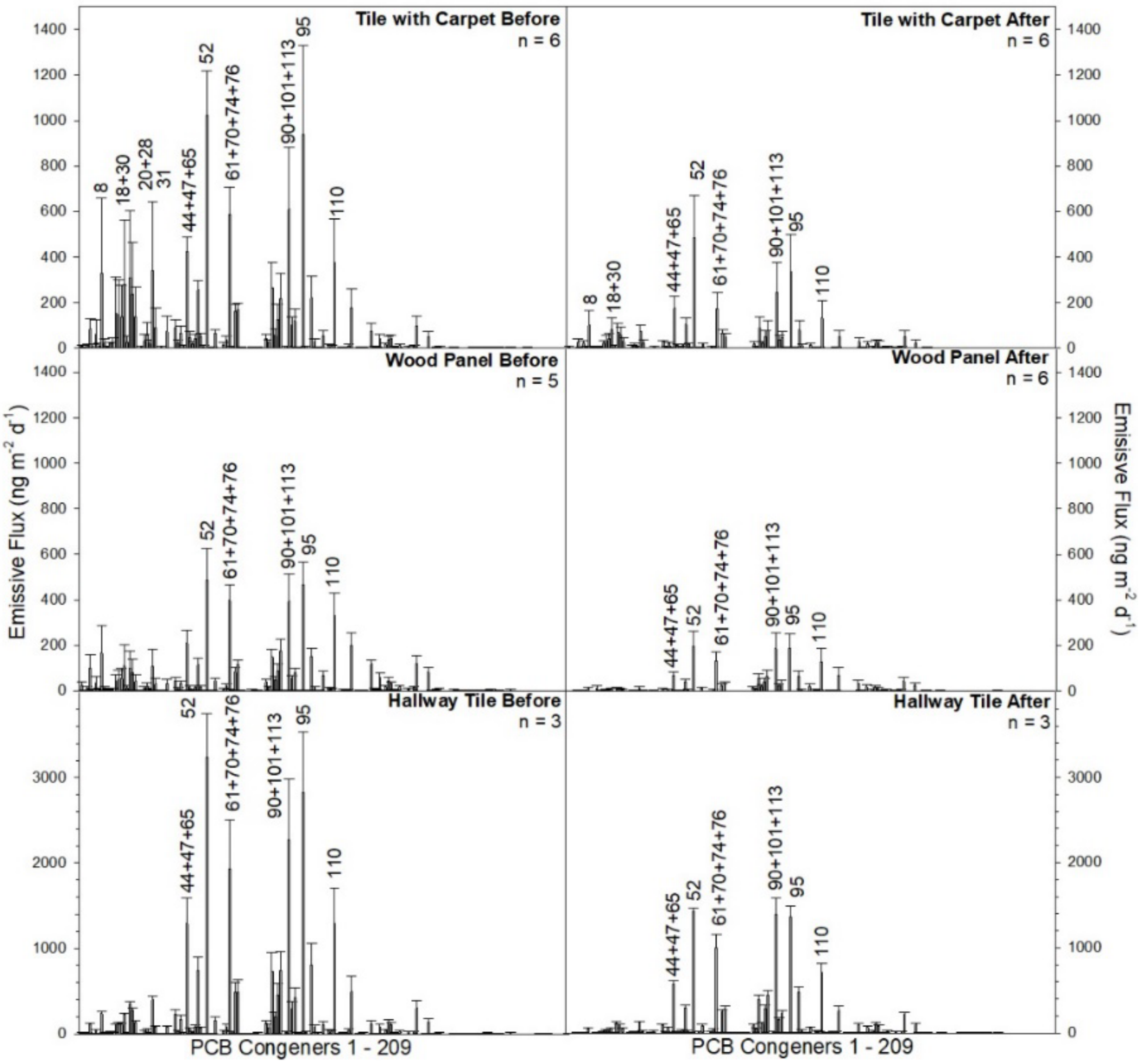

Figure S6. Average emissive flux (ng m^{-2} d^{-1}) of 171 individual and coeluting PCB congeners from tile overlaid with carpet (in rooms), wood panel, and bare tile (hallway) before and after hexane wiping. Error bars represent one standard deviation from the mean. Congeners with an average contribution plus one standard deviation above 5% of the total are labelled.

Table S13. The percent (%) contribution of each material emissions profile to the best-fit combination of source profiles correlated (cos θ) to a room air profile.

Room Number	1st Source	1st Source Contribution	2nd Source	2nd Source Contribution	Cos θ
132	Hallway Tile	67%	Room 132 Carpet + Tile	33%	0.98
137	Hallway Tile	92%	Room 137 Carpet + Tile	8%	0.97
140	Hallway Tile	86%	Room 137 Carpet + Tile	14%	0.97
171	Room 132 Carpet + Tile	63%	Hallway Tile	37%	0.99

For each room there was a combination of material emission profiles that best suited the room air profile (**Table S13**). The cos thetas for all best fit combinations of source emission profiles to room air profiles were above 0.97. Air profiles for Rooms 132, 137, and 140 were correlated to emissions from the hallway tile. All three rooms share the same hallway and construction date. Room 171 is not as influenced by the hallway tile profile as Rooms 132, 137, and 140. Room 171 is majority influenced by a source identical to Room 132 tile overlaid with carpet even though it is in a separate wing of the building as was constructed almost 10 years later. However, Room 132 is the room closest to the hallway that led to Room 171's wing of the building.

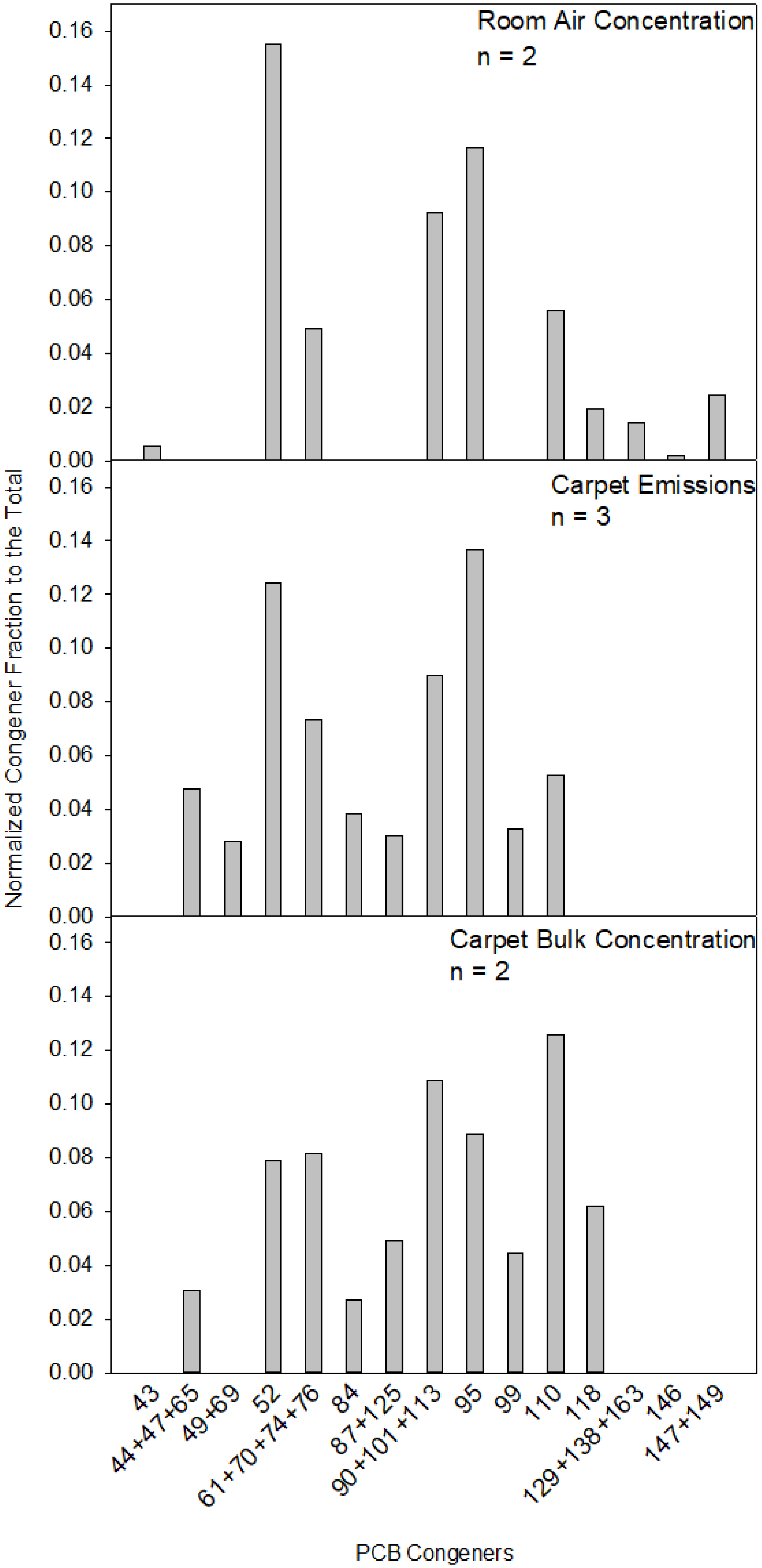
Room Air Concentration
n = 2
Carpet Emissions
n = 3
Carpet Bulk Concentration
n = 2
Normalized Congener Fraction to the Total
0.00
0.02
0.04
0.06
0.08
0.10
0.12
0.14
0.16
43
44+47+65
49+69
52
61+70+74+76
84
87+125
90+101+113
95
99
110
118
129+138+163
146
147+149
PCB Congeners

Figure S7. The normalized fraction of the total airborne concentration (n = 2), surface emissions (n = 3), and bulk concentration of 209 PCB congeners in air (n = 2). The 10 most dominant congeners (highest normalized fraction to the sample) are depicted.

Study Limitations

This study was conducted in unoccupied rooms which leads to more stagnant airflow than an occupied room. Increased airflow could increase emissions from surfaces.

References

(1) Bannavti, M. K.; Marek, R. F.; Martinez, A.; Hornbuckle, K. C. Dataset for Congener-specific Emissions from Floors and Walls Characterize Indoor Airborne PCBs. Iowa Research Online, 2023.
(2) Herkert, N. J.; Hornbuckle, K. C. Effects of room airflow on accurate determination of PUF-PAS sampling rates in the indoor environment. *Environ Sci Process Impacts* **2018**, *20* (5), 757-766. DOI: 10.1039/c8em00082d From NLM.
(3) Shoeib, M.; Harner, T. Characterization and comparison of three passive air samplers for persistent organic pollutants. *Environmental Science & Technology* **2002**, *36* (19), 4142-4151, Article. DOI: 10.1021/es020635t.
(4) Herkert, N. J.; Martinez, A.; Hornbuckle, K. C. A Model Using Local Weather Data to Determine the Effective Sampling Volume for PCB Congeners Collected on Passive Air Samplers. *Environmental Science & Technology* **2016**, *50* (13), 6690-6697. DOI: 10.1021/acs.est.6b00319.
(5) Lewis, R. G. Compendium of Methods for the Determination of Toxic Organic Compounds in Ambient Air. *United States Environmental Protection Agency* **1999**, *Compendium Method TO-10A* (EPA/625/R-96/010b).
(6) Bannavti, M. K.; Jahnke, J. C.; Marek, R. F.; Just, C. L.; Hornbuckle, K. C. Room-to-Room Variability of Airborne Polychlorinated Biphenyls in Schools and the Application of Air Sampling for Targeted Source Evaluation. *Environ Sci Technol* **2021**, *55* (14), 9460-9468. DOI: 10.1021/acs.est.0c08149 From NLM.
(7) Woolson, R. F. Wilcoxon Signed-Rank Test. *Wiley Encyclopedia of Clinical Trials* **2008**, 1-3, https://doi.org/10.1002/9780471462422.eoct979. DOI: https://doi.org/10.1002/9780471462422.eoct979 (acccessed 2022/05/21).
(8) Bannavti, M. K.; Jahnke, J. C.; Marek, R. F.; Hornbuckle, K. C. Dataset for Room-to-Room Variability of Airborne PCBs in Schools and the Application of Air Sampling for Targeted Source Evaluation. University of Iowa, 2021.
(9) Armstrong, R. A. When to use the Bonferroni correction. *Ophthalmic and Physiological Optics* **2014**, *34* (5), 502-508, https://doi.org/10.1111/opo.12131. DOI: https://doi.org/10.1111/opo.12131 (acccessed 2022/05/21).
(10) Rodenburg, L. A.; Meng, Q. Y. Source Apportionment of Polychlorinated Biphenyls in Chicago Air from 1996 to 2007. *Environmental Science & Technology* **2013**, *47* (8), 3774-3780, Article. DOI: 10.1021/es305024p.
(11) Herkert, N. J.; Jahnke, J. C.; Hornbuckle, K. C. Emissions of tetrachlorobiphenyls (PCBs 47, 51, and 68) from polymer resin on kitchen cabinets as a non-Aroclor source to residential air. *Environmental Science & Technology* **2018**, *52* (9), 5154-5160. DOI: 10.1021/acs.est.8b00966.
